# Symptoms of Attention Deficit Hyperactivity Disorder (ADHD) among adult eating disorder patients

**DOI:** 10.1186/s12888-016-1093-1

**Published:** 2017-01-17

**Authors:** Nils Erik Svedlund, Claes Norring, Ylva Ginsberg, Yvonne von Hausswolff-Juhlin

**Affiliations:** 1 Centre for Psychiatry Research, Department of Clinical Neuroscience, Karolinska Institute, Stockholm, Sweden; 2 Department of Medical Epidemiology and Biostatistics, Karolinska Institute, Stockholm, Sweden; 3 Stockholm Centre for Eating Disorders, att: Nils Erik Svedlund, Wollmar Yxkullsgatan 27 B, 11850 Stockholm, Sweden

**Keywords:** ADHD, Anorexia nervosa, Binge eating disorder, Bulimia nervosa, Comorbidity, Eating disorders, Prevalence, Purging

## Abstract

**Background:**

Very little is known about the prevalence of ADHD symptoms in Bulimia Nervosa and Binge Eating Disorder and even less in other eating disorders. This knowledge gap is of clinical importance since stimulant treatment is proven effective in Binge Eating Disorder and discussed as a treatment possibility for Bulimia Nervosa. The objective of this study was to explore the prevalence and types of self-reported ADHD symptoms in an unselected group of eating disorder patients assessed in a specialized eating disorder clinic.

**Methods:**

In total 1165 adults with an eating disorder were assessed with a battery of standardized instruments, for measuring inter alia ADHD screening, demographic variables, eating disorder symptoms and psychiatric comorbidity. Chi-square tests were used for categorical variables and Kruskal-Wallis tests for continuous variables.

**Results:**

Almost one third (31.3 %) of the patients scored above the screening cut off indicating a possible ADHD. The highest prevalence rates (35–37 %) were found in Bulimia Nervosa and Anorexia Nervosa bingeing/purging subtype, while Eating Disorder Not Otherwise Specified type 1–4 and Binge Eating Disorder patients reported slightly below average (26–31 %), and Anorexia Nervosa restricting subtype patients even lower (18 %). Presence of binge eating, purging, loss of control over eating and non-anorectic BMI were related to results indicating a possible ADHD. Psychiatric comorbidity correlated to ADHD symptoms without explaining the differences between eating disorder diagnoses.

**Conclusions:**

There is a high frequency of ADHD symptoms in patients with binge eating/purging eating disorders that motivates further studies, particularly concerning the effects of ADHD medication. The finding that the frequency of ADHD symptoms in anorexia nervosa with binge eating/purging is as high as in bulimia nervosa highlights the need also for this group.

## Background

Very little is known about the prevalence of Attention Deficit Hyperactivity Disorder (ADHD) symptoms in Bulimia Nervosa (BN) and Binge Eating Disorder (BED) and even less in other eating disorders (ED) [1]. This knowledge gap is of clinical importance, not least since stimulant treatment is proven effective in BED [2, 3] and discussed as a treatment possibility for BN [4, 5].

### Both ED and ADHD are common and serious disorders with longstanding debilitating symptoms

The lifetime prevalence of ED for females has been estimated to 0.9 % for Anorexia Nervosa (AN), 1.5 % for BN and 3.5 % for BED [6]. In a review article the mortality rate per 1000 person-years was 5.1 (95 % CI: 3.99–6.14) for AN, and 1.74 (95 % CI: 1.09–2.44) for BN; the 5-year recovery rates were 69 % for AN and 55 % for BN, respectively [7]. Females strongly outnumbers males in clinical samples for most ED diagnoses, except for BED where the sex ratio is less extreme [8]. Restricting ED dominate during childhood and bulimic in adulthood [9]. Furthermore there is a high comorbidity of other psychiatric disorders in ED [10, 11].

For ADHD, one meta-analysis estimated the prevalence to 5.9–7.1 % for children and adolescents and 5 % for young adults [12]. ADHD is classified into three types: hyperactive, inattentive, and combined. The inattentive type is more common among girls compared to boys [13]. In general, the course of ADHD includes a decrease in symptoms of hyperactivity and impulsivity, while inattention persists over time [14]. Detection and diagnosis tend to be later and less frequent in females than in males, probably related to the observed lower levels of extroversion and aggression in females [13]. Girls diagnosed with ADHD are also less likely to be treated with medication than boys [15]. The awareness among clinicians that ADHD exists in adulthood is also often limited [16]. The co-existence of ADHD and ED is thus easily overlooked. Due to the skewed gender distribution where females outnumbers males in ED, this could lead to a significant underestimation of ADHD in ED populations.

### There is an overlap in prevalence and treatment response between ADHD and ED

Regarding prevalence, a review concluded that females with ADHD had a higher risk of developing ED; BN rates found in ADHD ranged between 1 and 12 % versus 0–2 % in control groups [17]. In a cross-sectional study the prevalence of BED in ADHD was 8.1 % compared with 2.6 % in the general population [18]. Seitz et al. [5] found prevalence rates of former childhood ADHD to be 21 % in patients with BN versus 2.5 % in matched healthy controls. Blinder et al. [10] found co-morbid ADHD in 6 % of a large retrospective sample of 2436 female ED inpatients. Yates, Lund, Johnson, Mitchell, & McKee [19] found that 21 % of 189 inpatient females with ED reported at least six current ADHD symptoms. Wentz et al. [20] found that 17 % of a small sample (*n* = 30) of longstanding (mean 9.5 years; 95 % CI 6.2–12.8) ED fulfilled criteria of ADHD with both current and childhood symptoms. In a nationally representative sample, the occurrence of ADHD in young girls was associated with subsequent ED for females and with binging and/or purging behavior but not with restrictive behavior [21]. ED and ADHD share problems with impulsivity, depression, anxiety and low self-esteem and a causal chain has been proposed where the ADHD problems contribute to development of ED [22].

With respect to overlap in treatment response between ADHD and BN/BED, several case reports have observed a rapid and significant reduction of binge eating and purging symptoms when patients with coexisting BN/BED and ADHD were treated with stimulants [23–29]. Three possible explanations for this observed effectiveness of stimulant treatment for BN are discussed by Dukarm [25]: a) A common pathophysiology between ADHD and BN. One hypothesis is that dysregulated dopamine systems in both ADHD and BN/BED leads to risky behaviors (e.g. gambling, substance abuse or binge eating) to enhance the reward system. b) Untreated ADHD pave the way for BN (see above). and c) The appetite-suppressing effect of stimulants decreases the desire to binge and purge. In this case the effect of stimulants on BN/BED would be independent of a comorbid ADHD. Some support for this is found in a small double-blind placebo controlled study on 8 BN patients, without known ADHD, treated with intravenous methylamphetamine. Despite the small sample size methylamphetamine significantly reduced binge eating and purging [30]. In addition, in two randomized studies lisdexamfetamine reduced symptoms of binge eating in adults with BED, even while excluding those with previously known ADHD [2, 3].

Given the high prevalence and debilitating symptoms of bulimic ED and ADHD, and their substantial overlap, both in terms of prevalence and treatment response, it is important to further explore the relationship between these conditions. The lower likelihood of ADHD detection and stimulant treatment in females compared to males could also, in combination with the skewed gender distribution, contribute to increased prevalence rates of and impaired treatment results in bulimic ED. We examined the relationship between ED and ADHD symptoms in adults of both genders using prospectively collected quality register data covering the full range of ED diagnoses.

### Aim

One aim of this study was to explore the prevalence and types of self-reported ADHD symptoms in a large, unselected group of ED patients assessed in a specialized ED clinic. Another aim was to investigate the relations between ADHD symptoms and ED diagnoses and symptoms.

The specific questions addressed were: a) what is the prevalence of self-reported ADHD symptoms in severe ED? b) do the prevalence and types of self-reported ADHD symptoms differ between ED diagnoses and symptoms? and c) is the relationship between ADHD symptoms and ED diagnoses influenced by depressive, anxious or obsessive-compulsive symptoms?

## Methods

### Participants

**Fig. 1:**
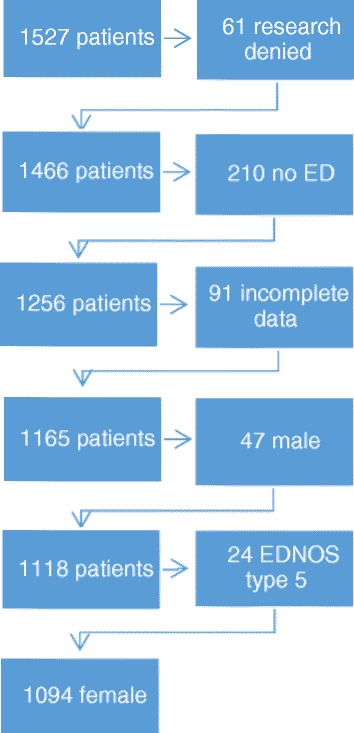
Patient flowchart

As displayed in Fig. 1, 1527 adult consecutive patients were seeking help at the Stockholm Centre for Eating Disorders (SCED) from 4 February 2013 through 18 September 2015. Of these, 61 did not consent to participate in research, 210 did not fulfil DSM-IV criteria for an eating disorder diagnosis and another 91 were excluded due to incomplete data. Of the remaining, 47 were males. Due to their documented differences in ADHD symptoms compared to females, with a risk of confounding the results, males are described separately and not included in the statistical analyses. The small (*n* = 24) Eating Disorder Not Otherwise Specified (EDNOS) type 5 group has different and unusual symptoms compared to all other ED diagnoses and was therefore also not included in the statistical analyses, thus leaving a total of 1094 female participants to analyze. Descriptive data including males and EDNOS type 5 are presented in Table 1.

**Table 1:** Demographic data across ED diagnoses and gender

ED and gender	Number	Age years Mean (SD)	BMI Mean (SD)	Living alone^b^ % (n/N)^a^	Work-/studying^c^ % (n/N)^a^
Females	1118	27.6 (8.6)	23.1 (6.0)	40.5 (394/973)	88.1 (966/1096)
AN-R	68	23.2 (6.6)	15.8 (1.4)	22.7 (15/66)	81.8 (54/66)
AN-BP	37	24.1 (6.2)	16.3 (0.9)	21.2 (7/33)	86.5 (32/37)
EDNOS-R	148	26.3 (8.1)	21.3 (5.8)	39.1 (54/138)	84.9 (124/146)
BN	421	27.6 (7.7)	23.8 (4.6)	45.1 (167/370)	90.2 (370/410)
EDNOS-BP	329	27.4 (8.1)	22.9 (4.6)	39.9 (110/276)	89.9 (294/327)
BED	91	34.2 (11.8)	33.0 (6.9)	51.5 (34/66)	83.0 (73/88)
EDNOS 5	24	29.8 (13.2)	20.1 (4.1)	29.2 (7/24)	86.4 (19/22)
Males	47	31.5 (10.9)	28.1 (9.9)	42.5 (17/40)	89.4 (42/47)
Total	1165	27.7 (8.7)	23.3 (6.3)	40.6 (411/1013)	88.2 (1008/1143)

^a^in-complete data, ^b^living alone or with own children (below 18 years age), ^c^working/studying on the open market

AN-R (Anorexia Nervosa restricting subtype), AN-BP (Anorexia Nervosa bingeing/purging subtype), EDNOS (Eating Disorder not otherwise specified), BN (Bulimia Nervosa purging and non-purging subtype), BED (Binge Eating Disorder = EDN 6)

SD (Standard deviation), *n* (number with criteria), *N* (Number in ED group analysed)

Participants were between 18 and 70 years of age (mean 27.7; SD 8.7) with BMI ranging from 11.6 to 60.4 (mean 23.3; SD 6.3). In total 41 % were living alone or with own children (below 18 years age) and 88 % were working or studying on the open market (Table 1).

### Measures

All participants were assessed with Stepwise, a web-based quality assurance system for clinical ED care [31]. From Stepwise we used data from the Structured Eating Disorder Interview (SEDI) [32] for ED diagnoses according to the DSM, fourth edition, Text Revision (DSM-IV TR). To facilitate the analyses we grouped the diagnoses as stringent as possible out from restricting, bingeing and purging symptoms, i.e. AN restricting subtype (AN-R), AN bingeing/purging subtype (AN-BP), BN purging and non-purging type (BN), EDNOS type 1–2 [EDNOS-R (R = restricting)], EDNOS type 3–4 [EDNOS-BP (BP = bingeing/purging)], EDNOS type 5, and BED that is classified as EDNOS type 6 in DSM-IV TR. SEDI also identifies individual patients with binge eating, purging and loss of control over eating. Preliminary validation of SEDI against the internationally extensively validated Eating Disorder Examination [33], interview has shown a concordance of 81 % for specific ED diagnosis and a Kendall’s Tau-b of 0,69 (*p* < 0.0001) [32]. Further, we used demographic data from Riksät, the national quality register for ED treatment [34]. From Stepwise we also used the Comprehensive Psychiatric Rating Scale (CPRS) a self-report instrument for depression, anxiety and obsession-compulsion [35]. The correlation between the self-ratings and interview ratings based on the Montgomery-Åsberg Depression Rating Scale and Brief Scale for Anxiety has been reported to be above 0.87 and 0.80 respectively [35]. For this study the screening version of the WHO ADHD Self-Rating Scale for Adults (ASRS-screener) for ADHD symptoms was added to Stepwise. This instrument consists of four questions addressing attention deficit symptoms and two questions for hyperactivity/impulsivity symptoms. Each question has five response alternatives; never, rarely, sometimes, often and very often, graded from 0 to 4 [36]. The total sum was used as the ASRS trait score (ASRSTS;range 0–24) and the dichotomized scale (0-13/14-24) for identifying possible cases of ADHD [37]. The four *attention deficit* and the two *hyperactivity/impulsivity* questions were also used separately as an inattention trait score (ITS; range 0–16) and a hyperactivity trait score (HTS; range 0–8) as proposed by Das, Cherbuin, Butterworth, Anstey & Easteal [38]. According to Kessler et al. [37], internal consistency reliability of the ASRS screener was in the range 0.63-0.72. In the current study, the Cronbach alpha coefficient for the ASRS screener was 0.82, with the subscales showing similar figures (ITS = 0.84 and HTS = 0.73).

### Statistical analysis

All analyses were performed with IBM SPSS Statistics version 23. Although the Kolmogorov-Smirnov statistics were significant for all continuous parameters they were all fairly normally distributed as concluded from histograms. Preliminary analyses were performed and revealed no major violation of assumptions of linearity, homogeneity of variances, homogeneity of regression slopes or homoscedasticity. Non-parametric tests were chosen since ASRS and CPRS do not fulfil criteria for an interval scale, and since group sizes for the different ED diagnoses varied considerably, which at least to some extent could compromise the assumption of normality. Chi-square tests for independence were used to analyze the dichotomized ASRS- (0-13/14-24) scale versus ED diagnoses and ED symptoms, respectively. Kruskal-Wallis tests were done to compare ASRS trait score, ITS, HTS and CPRS scores between ED diagnoses, with follow-up Mann–Whitney U tests with Bonferroni corrections to analyse group differences for ASRS trait score and CPRS between AN-R and bingeing/purging ED (AN-BP, BN, EDNOS-BP, BED). Spearman rho correlation analyses were done between the three CPRS scales (dep., anx. and com.) and ASRS trait score. All significance tests were two tailed; alpha-level of 5 %.

## Results

### Prevalence of self-reported ADHD symptoms

The prevalence of ASRS-screener results indicating a possible ADHD (ASRS trait score ≥14) was 31.6 % in females (*n* = 1094). For the groups not included in further analyses, corresponding results were 34.0 % for men and 12.5 % for patients belonging to the EDNOS type 5 group, resulting in a prevalence of ASRS-screener results indicating a possible ADHD of 31.3 % in the whole patient group (n = 1165). (Table 2).

**Table 2:** Clinical features

	Females^a^	Males	EDNOS 5
Number	1094	47	24
ASRS-screener ≥14 *n* (%)	346 (31.6)	16 (34.0)	3 (12.5)
ITS mean (SD)	6.95 (3.94)	7.47 (4.10)	6.17 (2.70)
HTS mean (SD)	3.85 (2.20)	3.96 (2.20)	3.42 (1.74)
Bingeing *n* (%)	740 (67.6)	36 (76.6)	10 (41.7)
Purging *n* (%)	544 (49.7)	17 (36.2)	4 (16.7)
Loss of control over eating *n* (%)	859 (78.5)	35 (74.5)	11 (45.8)
BMI > 17.5 *n* (%)	968 (88.5)	47 (100)	18 (75.0)
CPRS dep mean (SD)	10.56 (4.57)	9.97 (4.88)	9.17 (3.85)
CPRS anx mean (SD)	9.67 (4.19)	8.65 (4.54)	8.54 (3.30)
CPRS com mean (SD)	9.16 (4.25)	8.28 (4.78)	9.10 (4.47)

^a^excluding EDNOS 5. ASRS (The World Health Organization adult ADHD self-report scale)-screener (range 0–24), ITS (Inattention trait score; range 0–16), HTS (Hyperactivity trait score; range 0–8), CPRS (Comprehensive Psychiatric rating scale), dep (depression; range 0–27), anx (anxiety; range 0–27), com (obsession-compulsion; range 0–24)

### Differences in self-reported ADHD symptoms between ED diagnoses and symptoms

The highest frequency of possible ADHD was found in BN and in AN-BP, respectively. The EDNOS-BP, BED and EDNOS-R patients had intermediate frequencies and AN-R had the lowest. The difference in ASRS trait score ≥14 across ED diagnoses was significant with a small to medium effect size [*χ* ^2^ (5, *n* = 1094) = 15.30, *p* = 0.009, Cramer’s V = 0.12] (Table 3).

**Table 3:** ASRS-screener ≥14 across ED diagnoses in females

	AN-R	AN-BP	EDNOS-R	BN	EDNOS-BP	BED	Total	*p* ^a^	Cramer’s V
% (n/N)	17.6 % (12/68)	35.1 % (13/37)	25.7 % (38/148)	37.1 % (156/421)	31.0 % (102/329)	27.5 % (25/91)	31.6 % (356/1094)	<0.01	0,12

^a^Chi-square (two-sided), *n* (number with ASRS-screener ≥14), *N* (number in this ED group)

ASRS (The World Health Organization adult ADHD self-report scale)-screener (0-13/14-24)

AN-R (Anorexia Nervosa restricting subtype), AN-BP (Anorexia Nervosa bingeing/purging subtype), EDNOS (Eating Disorder not otherwise specified), BN (Bulimia Nervosa purging and non-purging subtype), BED (Binge Eating Disorder = EDNOS 6)

Kruskal-Wallis tests revealed statistically significant differences across ED diagnoses for ASRS trait score [*χ* ^2^ (5, *n* = 1094) = 31.89, *p* = 0.000], inattention trait score (ITS) [*χ* ^2^ (5, *n* = 1094) = 40.90, *p* = 0.000] and hyperactivity trait score (HTS) [*χ* ^2^ (5, *n* = 1094) = 12.84, *p* = 0.025].

Four Mann–Whitney U Tests each for ASRS trait score, ITS and HTS, were done pairwise comparing AN-R with all bingeing/purging ED groups (AN-BP, BN, EDNOS-BP and BED). This revealed significant differences for ASRS trait score in all tested pairs besides AN-R/AN-BP (*r* = 0.19-0.25), for ITS in all tested pairs (r = 0.19–0.32), and for HTS only between AN-R and EDNOS-BP (*r* = 0.13). ASRS trait score and ITS recorded highest median score in the BN group (*Md* = 12 and 8, respectively) and lowest median score in AN-R (*Md* = 7 and 5 respectively). HTS recorded the highest median score 5 in AN-BP and the lowest median score 3 in AN-R and BED (Table 4).

**Table 4:** Pairwise Mann–Whitney U Tests AN-R across binge-/purging ED and median scores across ED groups

		Median score	U	Z	*p*	r
AN-R	ASRSTS	7				
AN-BP (vs. AN-R)	ASRSTS	11	910	−2.34	0.019	0.23
BN (vs. AN-R)	ASRSTS	12	8942	−4.98	0.000*	0.23
EDNOS-BP (vs. AN-R)	ASRSTS	10	7922	−3.80	0.000*	0.19
BED (vs. AN-R)	ASRSTS	10	2187	−3.17	0.002*	0.25
AN-R	ITS	5				
AN-BP (vs. AN-R)	ITS	7	855	−2.72	0.007*	0.26
BN (vs. AN-R)	ITS	8	8480	−5.41	0.000*	0.24
EDNOS-BP (vs. AN-R)	ITS	6	7982	−3.73	0.000*	0.19
BED (vs. AN-R)	ITS	7	1930	−4.07	0.000*	0.32
AN-R	HTS	3				
AN-BP (vs. AN-R)	HTS	5	1033	−1.53	0.127	0.15
BN (vs. AN-R)	HTS	4	11801	−2.34	0.019	0.11
EDNOS-BP (vs. AN-R)	HTS	4	8995	−2.57	0.010*	0.13
BED (vs. AN-R)	HTS	3	2959	−0.47	0.635	0.04
AN-R	CPRS-dep	9.5				
AN-BP (vs. AN-R)	CPRS-dep	11.5	955	−2.03	0.042	0.06
BN (vs. AN-R)	CPRS-dep	11.0	11901	−2.23	0.026	0.07
EDNOS-BP (vs. AN-R)	CPRS-dep	10.0	9947	−1.44	0.150	0.04
BED (vs. AN-R)	CPRS-dep	10.0	2763	−1.16	0.248	0.04

* = sign. (when applying a Bonferroni adjustment of 0.05/4 = 0.013 to the alpha values)

ASRSTS (The World Health Organization adult ADHD self-report scale trait score; range 0–24), ITS (Inattention trait score; range 0–16), HTS (Hyperactivity trait score; range 0–8), CPRS-dep (Comprehensive Psychiatric rating scale for depression; range 0–27). AN-R (Anorexia Nervosa restricting subtype), AN-BP (Anorexia Nervosa bingeing/purging subtype), EDNOS-BP (Eating Disorder not otherwise specified bingeing/purging subtype), BN (Bulimia Nervosa purging and non-purging subtype), BED (Binge Eating Disorder = EDNOS 6)

When further exploring the association to ED symptoms, ASRS ≥14 was found to be positively related to binge eating [*χ* ^2^ (1, *n* = 1094) = 8.08, *p* = 0.004, Phi = 0.09], purging [*χ* ^2^ (1, *n* = 1094) = 8.52, *p* = 0.004, Phi = 0.09], loss of control over the eating [*χ* ^2^ (1, *n* = 1094) = 5.51, *p* = 0.019, Phi = 0.07] and BMI > 17.5 [*χ* ^2^(1, *n* = 1094) = 4.44, *p* = 0.035, Phi = 0.07].

### Influence of depressive, anxious or obsessive-compulsive symptoms on difference between ED diagnoses in self-reported ADHD

ASRS trait score had a medium strong positive correlation with the CPRS scales for depression (rho = 0.38, *p* <0.001), anxiety (rho = 0.42, *p* <0.001) and obsession-compulsion (rho = 0.45, *p* <0.001), respectively. Kruskal-Wallis tests revealed statistically significant differences across ED diagnoses for CPRS-dep. [*χ* ^2^ (5, *n* = 1094) = 13.15, *p* = 0.022] but not for CPRS-anx. (*p* = 0.245) or CPRS-com. (*p* = 0.208). Four Mann–Whitney U Tests for CPRS-dep. pairwise comparing AN-R with all bingeing/purging ED groups (AN-BP, BN, EDNOS-BP and BED) revealed no significant differences (Table 4).

## Discussion

### Prevalence of self-reported ADHD symptoms

In this consecutive series of adults seeking help at a large specialized ED clinic, the ASRS-screener was above cut-off (≥14), indicating a possible ADHD, in almost one third (31.3 %) of the entire patient group (*n* = 1165). This prevalence is high compared to estimates of ADHD frequency in ED in previous studies: 5.3–21 % [5, 10, 19, 20].

### Differences in self-reported ADHD symptoms between ED diagnoses and symptoms

A marked difference was seen in the frequency of ADHD symptoms between the ED diagnoses. The ASRS-screener indicated a possible ADHD about twice as often in BN patients as in AN-R. AN-BP had ASRS-screener levels almost as high as BN, which to our knowledge is not shown before. EDNOS-BP, BED and EDNOS-R patients had intermediate frequencies of ASRS-screener results above the cut-off level (≥14). Although AN-R had a low frequency indicating a possible ADHD in our study (17.6 %), it was still high compared to previous findings of 3 % [10] and also compared to ADHD prevalence among young adults in society 5 % [12]. A possible explanation of this high frequency might be that the ASRS-screener tends to overestimate symptoms of ADHD in this population. This underlines the need for a validation study of ASRS in an ED population. The current finding of a very high level of ASRS-screener scores indicating ADHD in AN-BP is in line with the observation in the study of a group with longstanding ED (mean 9.5 years; 95 % confidence interval 6.2–12.8) by Wentz et al. [20], that ADHD was found in AN-BP but not in AN-R. AN-BP is assumed to be prognostic unfavourable [39] and thus in great need for new treatment possibilities.

The analysis of ED symptoms indicated a significant positive relation between ASRS ≥14 and the presence of binge eating/purging behaviors, loss of control over eating and BMI > 17.5, however with small effect sizes limiting the interpretation of these findings. Our results were in accordance with a recent study, including a nationally representative sample, which showed that clinical ADHD was associated with clinical bingeing/purging [40].

Our finding that the ITS had a stronger relationship with ED than the HTS has previously been shown to be the case in adult ADHD where the hyperactivity/impulsivity symptoms are less prominent than among children and adolescents [19]. However both attention deficit and hyperactivity/impulsivity symptoms of ADHD are of importance for the comorbidity with ED. One possible explanation of this might be that loss of control is a central issue that co-varies with both ADHD and ED. Inattention can lead to confusion and impulsivity/hyperactivity can cause ill-considered actions, which both can contribute to loss of control. This possible common factor seems to be primarily associated with binge eating and purging behaviors. In clinical work it is also obvious that AN patients, despite the primary impression of extreme control over the eating, tend to themselves experience a more or less total uncertainty about the adequacy of their food intake, due to their anorectic obsessions, resulting in loss of control.

### Influence by depressive, anxious or obsessive-compulsive symptoms on difference between ED diagnoses in self-reported ADHD

The medium strong correlations between the ASRS trait score and depression, anxiety and obsession-compulsion respectively, estimated with CPRS, are in line with the high psychiatric comorbidity for depression and anxiety in both ADHD [41] and ED [10, 11]. However, for the three CPRS variables, there were significant differences across ED diagnoses only for CPRS-depression and pairwise comparisons between AN-R and the four bingeing/purging ED diagnoses (AN-BP, BN EDNOS-BP and BED) did not show significant differences for CPRS depression. Accordingly, the CPRS variables cannot fully explain the differences in ASRS trait score between ED diagnoses.

### Males and EDNOS 5

There were too few males and females with an EDNOS type 5 diagnosis in the study to include them in any meaningful statistical analyses. However, it seems as if the frequency of possible ADHD among men is on the same level as among the bingeing/purging females, while it seems to be much lower than in any other group among the EDNOS type 5 patients.

### “Knowledge and knowledge gaps”

ED and ADHD share many symptoms [22] and the twofold differences in ASRS screener results between ED diagnoses indicate that these shared symptoms are mainly related to bingeing and purging ED symptoms. Effect sizes for these differences were small to medium supporting an assumption of other differentiating factors besides symptoms shared with ADHD.

There is a great need to conduct research aimed at better understanding the nature of the association between ADHD and ED. Because of the methodological limitations, it would also be very useful if this study could be replicated using more detailed assessments of ADHD symptoms in as large a sample as possible.

There are cumulating evidence of a good effect on bulimic symptoms when comorbid ADHD is treated with stimulants [23–29]. In addition, two recently published randomized multicenter studies showed that the stimulant lisdexamfetamine reduced symptoms of binge eating in adults with BED, even while excluding those with previously known ADHD diagnosis [2, 3]. In the study by Bleck et al. [40] subclinical ADHD, both inattentive and hyperactive/impulsive, was associated with subclinical bingeing/purging but not with restricting behaviors. Together this raises the question whether degree of ADHD symptoms, confirmed ADHD diagnosis or actual ED symptoms are the most important parameters to guide clinical decisions concerning stimulant medication in adult ED patients. This motivates further randomized trials with stimulant treatment for bingeing/purging ED-patients with and without a concomitant ADHD diagnosis. The finding of ADHD symptoms in AN-BP as high as in BN evoke the question if it is possible to try stimulant medication also in this group, if proven effective for BN. However the risk due to appetite suppression in an underweight patient must be taken into consideration. Another possible approach for deepened understanding could be a longitudinal study to investigate how treatment for ED interacts with ADHD symptoms and vice versa.

### Strengths and limitations

There are several strengths with this study to mention. First, the cohort constitutes the total intake of adult patients in a single clinic for a certain period of time. Second, the study sample is comparably large. Third, the study sample is recruited during an extended time period (31 months) minimizing confounding from time dependent differences. Fourth, there is no demand for an admission note. Fifth, men are included. Altogether, we suggest these results to be representative for adult patients searching help at a specialized ED clinic.

However, there are some limitations of this study to consider. First, an ADHD diagnosis postulate the presence of ADHD symptoms since childhood. The ASRS scale used in the present study only records ADHD symptoms during the last six months, probably resulting in an overestimation of ADHD prevalence. Second, the use of a very short rating scale for ADHD is unlikely to identify the full range of symptoms associated with this condition. Third, the use of web-based self-report instruments made it possible to examine a large cohort of patients. Something that had been difficult had we chosen more time-consuming diagnostic methods. However, this also means that our results suffer from the validity problems that accompany the use of self-report instruments. Fourth, in a help seeking population like the present one there could be a bias to overestimate symptoms. We have not controlled for the possibility that such a bias is unevenly distributed in the different ED diagnoses and thus distorting our results. Fifth, the assessment methods used do not supply information that makes it possible to control for other possible reasons for ADHD-like symptoms, like personality or dissociative disorders, learning disability or medical reasons. Thus, it is not possible to rule out such symptoms as alternative explanations to our results. Sixth, the cross sectional design of the study mean that we cannot establish a causal link between ADHD symptoms and ED. Seventh, there is a shift from restricting to bulimic ED diagnoses and also a symptom shift in ADHD with increasing age, meaning that our results may not be applicable for children and adolescents. Eighth, the number of men were too small for sub group statistics.

## Conclusions

This prospective study, including a much larger patient cohort than previous studies, supports earlier findings of a high frequency of ADHD symptoms across separate ED diagnoses, especially those with bingeing/purging behaviors. The finding that patients with the bingeing/purging subtype of AN have ASRS-results indicating a possible ADHD on level with BN patients is new and in need of replication. The study also lends further support to recent observations of inattentive ADHD symptoms being more prominent than hyperactivity/impulsivity symptoms in adult female ED patients. Patients with BN, BED, bulimic EDNOS and AN bingeing/purging subtype might benefit from a better understanding of the connections between their ADHD symptoms and their ED. Further studies on the implications for this comorbidity including controlled treatment trials with stimulants for bingeing/purging ED are needed.

## Abbreviations

ADHD: Attention deficit hyperactivity disorder
AN: Anorexia nervosa
anx.: Anxiety
ASRS: WHO adult ADHD self-rating scale
BED: Binge eating disorder
BMI: Body mass index
BN: Bulimia nervosa
BP: Bingeing/purging
com.: Compulsive
CPRS: Comprehensive psychiatric rating scale
dep.: Depression
DSM-IV TR: Diagnostic and statistical manual of mental disorders 4^th^ edition text revision
ED: Eating disorders
EDNOS: Eating disorders not otherwise specified
HTS: Hyperactivity trait score
ITS: Inattention trait score
R: Restricting
SCED: Stockholm centre for eating disorders
SEDI: Structured eating disorder interview
